# ErythroCite: a database on red blood cell size of fishes

**DOI:** 10.1038/s41597-026-06904-1

**Published:** 2026-02-27

**Authors:** Félix P. Leiva, Rafael Molina-Venegas, Katharina Alter, Carolina A. Freire, A. Jan Hendriks, Adam Hermaniuk, Léon Serre-Fredj, Milad Shokri, Marcin Czarnoleski, Felix C. Mark

**Affiliations:** 1 Integrative Ecophysiology, Alfred Wegener Institute Helmholtz Centre for Polar and Marine Research, 27570 Bremerhaven, Germany; 2 Department of Environmental Science, Radboud Institute for Biological and Environmental Sciences, Radboud University Nijmegen, 6500 GL Nijmegen, The Netherlands; 3 Terrestrial Ecology Group (TEG-UAM), Departamento de Ecología, Universidad Autónoma de Madrid, Madrid, Spain; 4 Centro de Investigación en Biodiversidad y Cambio Global (CIBC-UAM), Universidad Autónoma de Madrid, Madrid, Spain; 5 Estación Biológica de Doñana (EBD - CSIC), 41092 Sevilla, Spain; 6 Department of Coastal Systems, Royal Netherlands Institute for Sea Research, PO Box 59, 1790 AB Den Burg, Texel The Netherlands; 7 Department of Marine Biology, Institute of Biological Sciences, University of Rostock, Albert Einstein Straße 3, 18057 Rostock, Germany; 8 Departamento de Fisiologia, Setor de Ciências Biológicas – Centro Politécnico, Universidade Federal do Paraná, Curitiba, Paraná Brazil; 9 Life History Evolution Group, Institute of Environmental Sciences, Faculty of Biology, Jagiellonian University, Gronostajowa 7, 30-387 Kraków, Poland; 10 Department of Evolutionary and Physiological Ecology, Faculty of Biology, University of Białystok, Ciołkowskiego 1J, 15-245 Białystok, Poland; 11 Laboratory of Ecology, Department of Biological and Environmental Sciences and Technologies, University of Salento, 73100 Lecce, Italy; 12 National Biodiversity Future Center (NBFC), 90133 Palermo, Italy

**Keywords:** Evolutionary ecology, Taxonomy

## Abstract

Size is a fundamental trait in biology, and cell size plays a key role in cellular functions, influencing physiological adaptations and evolutionary processes in living organisms. For decades, scientists have been fascinated by the considerable variation in cell sizes among animals, yet systematic efforts to compile such data have been scarce. To address this gap, we employed a systematic map approach to create ErythroCite, an open-source database of fish erythrocyte sizes. This comprehensive resource encompasses 1,764 records from 660 species among four major lineages: Actinopterygii, Chondrichthyes, Dipnoi, and Cyclostomata. Our findings reveal a remarkable 414-fold range in cell volume, with most studies on bony fishes and limited data on juveniles and earlier life stages. Life stage and sex were infrequently reported, but available data showed equal representation of adult of females and males. ErythroCite offers valuable insights for studies in macroecology, macrophysiology, comparative physiology, evolutionary biology and cell biology. We anticipate this resource will facilitate comparative approaches and meta-analyses, globally driving further exploration of erythrocyte diversity and function in fish.

## Introduction

Size plays a pivotal role in biology due to its profound impact on the functioning of life^1^. For centuries, scientists have been fascinated by the causes and consequences of size-related variations among organisms. Although much of this interest has centred on overall body size, the exploration of cellular characteristics can be traced back to 1675, when the description of human red blood cells was provided^2,3^. Two centuries later, in 1875, George Gulliver’s illustrations revealed a remarkable variation in sizes of blood cells across vertebrates^4^. Gulliver’s work enhanced the understanding of erythrocyte diversity within the animal kingdom, particularly regarding characteristics associated with the variation in size of erythrocytes.

In most vertebrates, erythrocytes, or red blood cells (RBCs), represent the predominant blood cell type and the most abundant cellular component, playing a central role in metabolic physiology^5^. Their functionality stems primarily from haemoglobin, a specialised oxygen- and carbon dioxide-binding protein that facilitates the physiological process of oxygen delivery from respiratory organs to tissues^6,7^. Moreover, erythrocyte characteristics provide insights into how species have physiologically adapted to different environmental conditions^8–11^. Recent studies have demonstrated that cell size significantly influence ectothermic species’ responses to rising ambient temperatures^12–15^. Additionally, studies indicate intercorrelations between cell sizes across various organs and tissues^16^. This systemic relationship positions erythrocyte size as a simple yet useful proxy for assessing whole-organism cellular dimensions^17,18^. However, despite the importance of cell size in monospecific studies and multi-species comparison on other vertebrate groups^19^, there is currently no comprehensive, up-to-date database on RBC characteristics across diverse species.

To address this knowledge gap, we developed ErythroCite, the most extensive database of cell size-related traits to date, incorporating data for 660 fish species. ErythroCite goes beyond merely cataloguing blood cell size by integrating phylogenetic relationships, biological traits, and ecological information of four lineages of fishes. We focused on fish as a starting point for several reasons. First, fish account for approximately 50% of all vertebrate species, with over 35,000 described species^20^. Their erythrocytes are distinctively oval, flattened, and biconvex in shape^21^. Unlike the enucleated red blood cells of mammals, these nucleated cells offer valuable insights into the evolutionary adaptations of other ectothermic vertebrates, such as amphibians and reptiles. Second, while both aquatic organisms and high-altitude terrestrial vertebrates face oxygen limitations, water-breathing species, such as fishes, are more frequently exposed to low and fluctuating oxygen condition. Consequently, their gas transport systems-including the properties of red blood cells-must function efficiently to ensure oxygen delivery to tissues.

Third, the availability of trait databases for fish enables integration of ErythroCite with other datasets, enhancing our understanding of factors influencing variations in cell size. Finally, the establishment of a red blood cell database for fish is necessary to enhance and update existing initiatives, such as the Animal Genome Size Database^22^, which includes cell size information for several vertebrates groups. This should be achieved through a systematic, multilingual approach to literature review and data collection.

We expect ErythroCite helps researchers to conduct more robust comparative analyses and investigate the adaptive significance of erythrocyte size across a diverse range of fish species, thereby facilitating a deeper understanding of its evolutionary importance. In particular, we anticipate that the creation of this database will strengthen the current theory of optimal cell size^9^, which relates cell size to metabolism of organisms.

## Methods

We follow MeRIT guidelines established by Nakagawa *et al*.^23^ to ensure better clarity and transparency in our reporting and description of methods. These guidelines use author initials in the methods section to attribute specific tasks to individual contributors, complementing the Contributor Roles Taxonomy system (CRediT, https://credit.niso.org/).

### Literature searches

Our objective was to compile comprehensive data on the cytomorphology of red blood cells in fish species. Specifically, we identified studies that quantified parameters such as cell area and volume, as well as nuclear area and volume. Additionally, we collected associated geographical, biological, and ecological metadata for each entry and species. Furthermore, we gathered bibliometric information for each study for literature mapping on this subject.

**Fig. 1:**
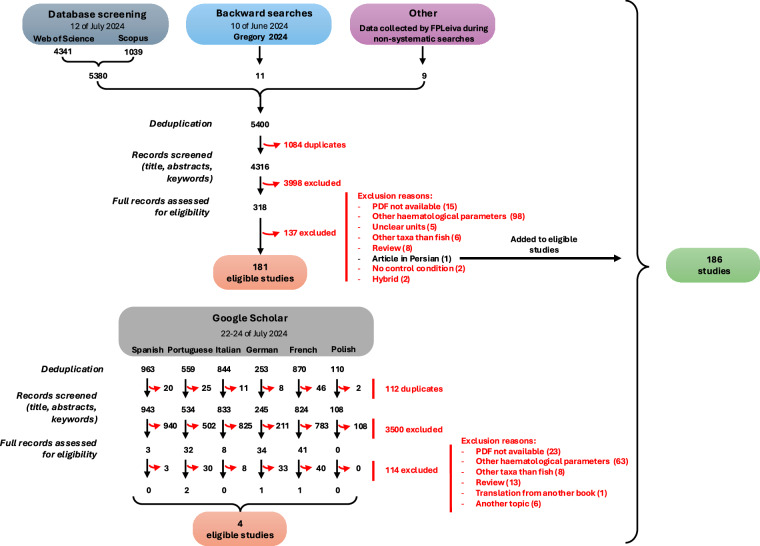
PRISMA-type diagram showing the systematic literature search for studies reporting cell size measurements in fish red blood cells. For each screening and exclusion stage, the number of studies is detailed. The diagram is based on a previous study by Pottier *et al*.^264^.

The search for information was conducted by FPLeiva using three search engines: ISI Web of Science (core collection), Scopus, and Google Scholar (Fig. 1). The first two search engines were used exclusively for searches in English on 12 July 2024, utilising Radboud University’s subscription to these services. The combination of Boolean search terms employed was: (red blood cell* OR erythrocyte OR RBC OR haematids OR red corpuscle* OR erythroid) AND (area OR size OR dimension OR volume OR diameter OR morpho*) AND (fish OR teleost OR shark* OR ray* OR skate OR ratfish OR ghostshark OR spookfish OR aquatic vertebrate OR elasmobranchii OR chondrichthyes OR osteichthyes OR ray-finned fish* OR bony fish*). From these searches, the full records were downloaded, including abstracts, keywords, and all relevant information, across all years and editions, and document types. Using ISI Web of Science, a total of 4,341 records were identified, whilst in Scopus, 1,039 records were found.

The search using Google Scholar was conducted on 22–24 July 2024, targeting studies published in Spanish, Italian, Portuguese, German, French, and Polish. To facilitate this multilingual search, we translated the English keywords into these six languages. For all languages except Spanish (the native language of FPLeiva), we used DeepL (www.deepl.com/) for initial translations. Native speakers then verified the accuracy of these translations: CAFreire for Portuguese, MShokri for Italian, KAlter for German, LSerre-Fredj for French, and AHermaniuk for Polish. We chose these languages to optimize the inclusion of non-English studies that could be read by at least one of the manuscript’s authors. The software Publish or Perish^24^ was used to search and extract records for each language. To accommodate Google Scholar’s 256-character search string limit, we modified our initial Boolean terms for each language. We condensed the search strings while preserving the essential concepts of our research question, ensuring comprehensive searches across all target languages despite Google Scholar’s constraints. Table 1 provides detailed translations of these modified search strings.

**Table 1:** Keywords combination used to search for references in seven different languages.

Language	Keywords combination
English	(red blood cell* OR erythrocyte OR RBC OR haematids OR red corpuscle* OR erythroid) AND (area OR size OR dimension OR volume OR diameter OR morpho*) AND (fish OR teleost OR shark* OR ray* OR skate OR ratfish OR ghostshark OR spookfish OR rabbitfish OR aquatic vertebrate OR elasmobranchii OR chondrichthyes OR Osteichthyes OR ray-finned fish* OR bony fish*)
Spanish	(glóbulo rojo* OR eritrocito OR hematíes OR corpúsculo rojo* OR eritroide) AND (área OR tamaño OR dimensión OR volumen OR diámetro OR morfo*) AND (pez OR peces OR teleósteo OR tiburón OR raya* OR quimera OR vertebrado acuático OR elasmobranquios OR chondrichthyes OR osteichthyes OR pez óseo)
Portuguese	(glóbulo vermelho* OR eritrócito OR hematídeos OR corpúsculo vermelho* OR eritroide) AND (área OR tamanho OR dimensão OR volume OR diâmetro OR morfo*) AND (peixe OR teleósteo OR tubarão* OR raia* OR quimera OR vertebrado aquático OR elasmobrânquios OR chondrichthyes OR osteichthyes OR peixe ósseo*)
Italian	(globulo rosso OR eritrocita OR ematidi OR corpuscolo rosso OR eritroide) AND (area OR dimensione OR volume OR diametro OR forma) AND (pesce OR teleosteo OR squalo* OR razza OR chimera OR vertebrato acquatico OR elasmobranchi OR chondrichthyes OR osteichthyes OR pesce osseo)
French	(globule rouge OR érythrocyte OR hématides OR globule rouge OR érythroïde) AND (zone OR taille OR dimension OR volume OR diamètre OR morpho) AND (poisson OR téléostéen OR vertébré aquatique OR élasmobranches OR chondrichtyens OR ostéichthyens OR poisson osseux)
German	(rotes Blutkörperchen* OR Erythrozyt OR rote Blutzellen* OR erythroid) AND (Bereich OR Größe OR Dimension OR Volumen OR Durchmesser OR Morphologie) AND (Fisch OR Teleostei OR aquatisches Wirbeltier OR chondrichthyes OR osteichthyes OR Knochenfische)
Polish	(czerwona krwinka* OR erytrocyt* OR hematydy OR czerwony krwinek* OR erytroid) AND (obszar OR rozmiar OR wymiar OR objętość OR średnica OR morfo*) AND (ryba OR teleost OR rekin OR płaszczka OR kręgowiec wodny OR elasmobranchii OR chondrichthyes OR osteichthyes OR oścista ryba)

The non-English searches are not an exact word-to-word translation due to some limitations of Google Scholar.

The Google Scholar searches conducted across various languages yielded a total of 3,599 studies. In total, our multi-engine, multilingual search produced 8,979 records. Subsequently, we screened these records to eliminate duplicates and evaluated their relevance based on titles, abstracts, and keywords.

In addition to our systematic searches, we employed complementary strategies to improve our literature search. For backward searches, we used a subset of the Animal Genome Size database^22^ (http://www.genomesize.com/cellsize/fish.htm) as a starting point. On the 10 of June 2024, FPLeiva accessed the latest version of this database and identified eleven relevant studies. Furthermore, FPLeiva has been compiling information on cell sizes of various ectotherm clades, including fish, through non-systematic searches. This ongoing effort added nine more studies to our review (Fig. 1).

To streamline the screening process, we utilized Rayyan^25^, an artificial intelligence-based platform designed to expedite systematic reviews by reducing the time required for each screening step. The screening was conducted by different team members based on their language expertise: FPLeiva handled the Spanish and English records, while CFreire screened Portuguese studies. MShokri was responsible for Italian, KAlter for German, LSerre-Fredj for French, and AHermaniuk for Polish studies.

### Eligibility criteria

We applied the following inclusion criteria: (i) only primary research articles were included, ensuring original data and appropriate credit to primary sources; (ii) we focused on species-specific data for consistency and comparability, excluding genus-level data and hybrid species; only studies measuring mature erythrocytes were considered, avoiding those including immature or developing cells; (iii) we selected studies working with diploid organisms, excluding polyploids due to potential cell size variations from different chromosomal loads^26^, though we noted as comments when additional data were also available for polyploids; (iv) in cases involving various treatments, only studies that reported experimental control conditions as labelled in the study were considered, to ensure the results were comparable across studies in ErythroCite; (v) for the few instances in which anticoagulants were used during blood collection, we used the mean cell size, as anticoagulants can influence these measurements^27^; (vi) and when several techniques to obtain cell sizes were employed, we prioritised data obtained from blood smears, as they provide a more consistent measure of cell size compared to live cells, which can vary in size due to their physiological state. Using these inclusion criteria, the number of studies included in ErythroCite across all languages was 186, which were all cited here^4,28–212^.

### Data extraction and metadata

We endeavoured to incorporate the direct estimates of cell area, cell volume, mean corpuscular volume, nucleus area and nucleus volume from the original studies as much as possible. However, in numerous studies, only the lengths of the major and minor axes of the cells and their nuclei were reported. When this was the case, we employed standard formulae to calculate the area and the volume of the cell or its nucleus, assuming that both the cell and its nucleus were shaped like ellipsoids or oblate spheroids^22,213^.

The formula for cell area (*A*) was:

$$A=\pi \times \left(\frac{a}{2}\right)\times \left(\frac{b}{2}\right)$$

The formula used for cell volume (*V*):

$$V=\frac{4}{3}\times \pi \times \left(\frac{a}{2}\right)\times {\left(\frac{b}{2}\right)}^{2}$$

Where ‘a’ and ‘b’ denote the lengths of the semi-major and semi-minor axes of an ellipse, respectively. These parameters were employed in the preceding equations to calculate the area (*A*) and volume (*V*) of erythrocytes modelled as elliptical shapes.

While most methods for measuring cell volume rely on fixed blood smears, alternative approaches exist. Various studies have reported mean corpuscular volume (MCV, measured in μm³) as a proxy for cell volume. MCV is typically estimated using a standard formula, as reviewed by Witeska *et al*.^21^:

$${MCV}=\frac{{Ht}\times 10}{{RBC}}$$

Where Ht is the haematocrit and RBC is the red blood cells counts.

In our database, MCV values are presented in a separate column and should be interpreted with caution when compared to cell volume measurements obtained from blood smears, as emphasized by Gregory^22^. This distinction is important because MCV is derived from haematological parameters, while smear measurements are obtained through direct microscopic observation. Moreover, MCV represents an average value for the entire erythrocyte population, whereas cell volume estimates from smears provide measurements of individual cells.

Despite the extensive number of studies included in this work, the collection of methodological information (metadata) related to cell size estimation was relatively limited. Nevertheless, we gathered metadata associated with collection location where the species were sourced, body size, sex, and life stage studied. When location descriptions were general (e.g., Araucanía Region, Chile), coordinates were obtained from the OpenStreetMap Data Search Engine Nominatim (http://nominatim.openstreetmap.org). For more specific locations, such as named hatcheries, institutes, or localities, we utilized Google Maps to determine precise geographical positions. Additionally, we provided, in an additional column, the description of the location from where the animals were sourced, which should be used to filter, for example, wild-collected animals, in case users are interested in testing latitudinal hypotheses of cell size variation. This is because, for instance, an institute location does not necessarily correlate with natural habitat conditions in the same area.

We converted fish body sizes reported in length units to wet body mass (in grams) using species-specific length-weight relationships obtained from FishBase^20^. There was a single study, Martins *et al*.^127,214^, providing approximately 3,700 observations for 15 fish species. For this study, we averaged the cell sizes at the specimen level (five individuals per species). For studies presenting cell size data exclusively in figures without accompanying textual or tabular information, we employed Plot Digitizer, a Java-based program designed to extract X-Y coordinates from graphs (http://plotdigitizer.sourceforge.net).

### Taxonomy and phylogeny

The species names were scrutinised for synonyms and any updates that might influence the taxonomy. To accomplish this, we adopted the taxonomic harmonisation procedure outlined by Lenoir *et al*.^215^ and Leiva *et al*.^216^. This taxonomic harmonization consists of three automated steps: first, we searched for species names in the National Center for Biotechnology Information (NCBI) taxonomy database; second, we verified any unmatched taxonomic entities using the Integrated Taxonomic Information System (ITIS) database; and third, we cross-checked remaining unmatched entities against the Global Biodiversity Information Facility (GBIF) database. If a match was identified, the corrected taxonomic entity was re-evaluated through the entire verification process in NCBI and ITIS to ensure accurate classification. Ultimately, only names at the species levels were retained in the database, with subspecies aggregated at the species level (e.g., *Catostomus catostomus*). The majority (91%) of species name verifications were sourced from NCBI, with ITIS and GBIF providing additional support. For the remaining species that could not be verified through this process, manual checks were performed using additional resources such as FishBase^20^ and World Register of Marine Species (WoRMS)^217^. When using ITIS, several species were grouped within the class Teleostei, while GBIF left most species unassigned to any class. In these cases, we manually reassigned these species to the class Actinopterygii. To address potential issues of data interoperability, we have additionally included the taxonomy of the species based on FishBase. This will allow users to more easily combine the cell size data with other fish traits, thereby enhancing interoperability between datasets from different studies^218^.

We retrieved the phylogenetic relationships of the species from Open Tree of Life (OTL)^219^. For *Choerodon albigena*, which lacked information in the OTL, we added it using the phylogenetic position of its sister species, *Choerodon cephalotes*.

We utilised the harmonised species list to obtain the associated realm for each species from FishBase^20^, accessed through WoRMS on 14 November 2024, using the WoRMS Taxon Match tool^220^. In WoRMS, the realms freshwater, brackish, marine, and terrestrial are assigned as a binary variable (1 or 0). In our database, we recorded whether a species occupies more than one aquatic realm throughout their life. This process resulted in five categories: marine, marine-brackish-freshwater, marine-brackish, freshwater-brackish, and freshwater, reflecting the diversity of habitats that species occupy and recognising their ability to adapt to different environmental conditions throughout their life cycle.

All analyses were carried out in R version 4.3.1^221^. The *rutils* package version 0.0.0.9^222^, *readxl* package version 1.4.3^223^, *dplyr* package version 1.1.4^224^, *plyr* package^225^, *writexl* package version 1.5.0^226^, *tibble* package version 3.2.1^227^, *sessioninfo* package version 1.2.2^228^, *rnaturalearth* package version 1.0.1^229^, *tidygeocoder* package version 1.0.5^230^ *kableExtra* package version 1.4.0^231^ and *DataExplorer* package version 0.8.3^232^ were used to curate, format, and inspect data. The *RefManageR*^233,234^ was used to manipulate references. The *rgbif* package version 3.7.8^235,236^, *rfishbase* package^237^ and *taxize* package version 0.9.98^238,239^ were used for the taxonomic harmonization. The *rotl* package version 3.1.0^240^, *ape* package version 5.8^241^, *phytools* package version 2.1-1^242^ and *ggtree* package^243–247^ were used to create and manipulate phylogenetic trees. The *ggplot2* package version 3.5.1^248^, *ggpubr* package version 0.6.0^249^, *fishualize* package version 0.2.3^250^, *cowplot* package version 1.1.3^251^ and *ggthemes* package version 5.0.0^252^ were used to produce figures.

## Data Records

All materials, including the database, R code, and additional supplementary content are available under the Creative Commons Attribution 4.0 International licence (CC BY 4.0). ErythroCite is archived on GitHub at https://github.com/felixpleiva/ErythroCite and preserved on Zenodo^253^. This repository contains the data, metadata, and R code (https://felixpleiva.github.io/ErythroCite/) used for data curation, as well as for generating the figures and phylogenetic tree. References are also provided as a BibTeX file. ErythroCite will be updated as necessary to incorporate new studies and any identified corrections. In all cases, updates will comply with the standards of the Semantic Versioning Specification (SemVer, https://semver.org/).

## Data Overview

**Fig. 2:**
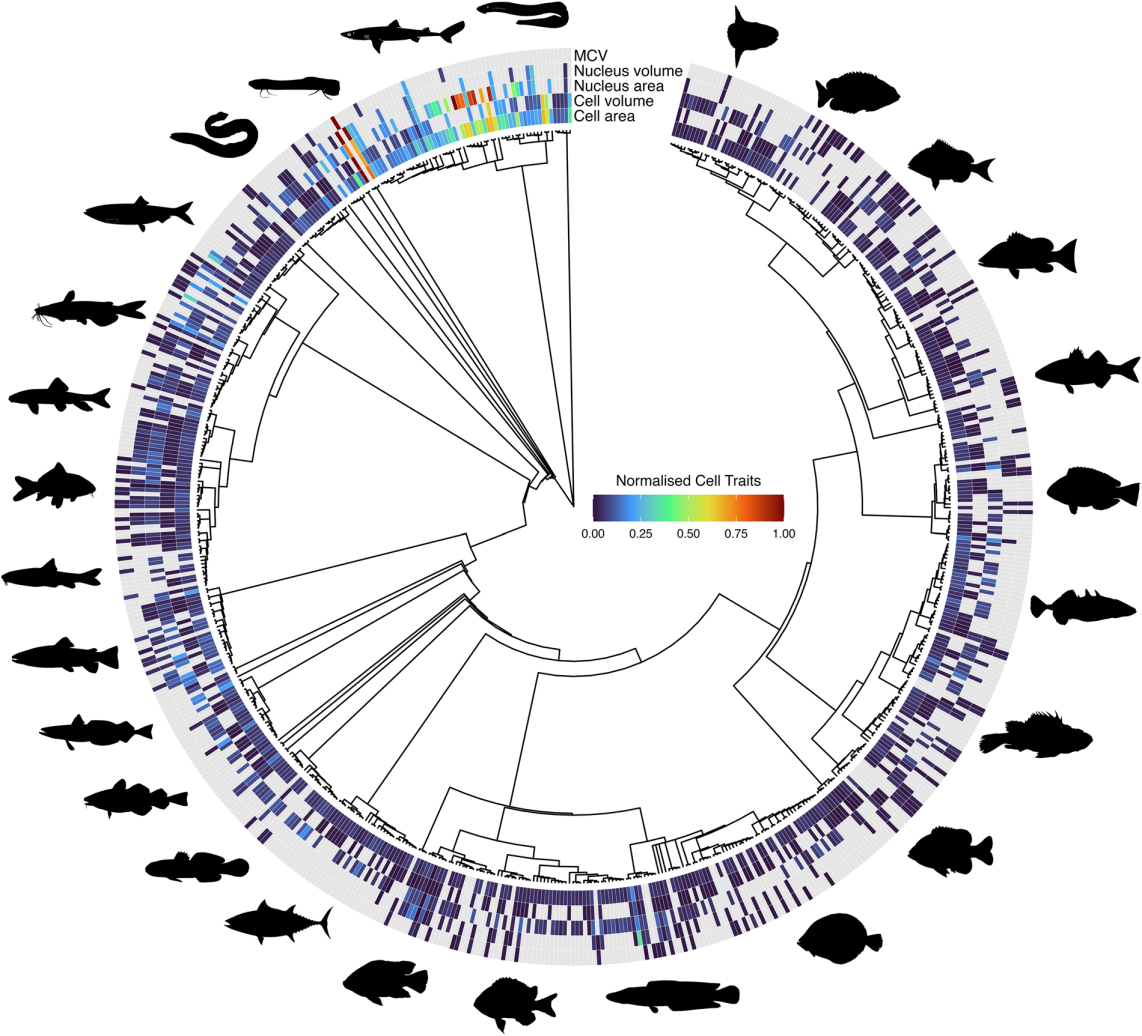
Phylogenetic relationships and cell size trait distribution among 629 fishes. For illustrative purposes only, the trait values were averaged by species and then normalised by subtracting the minimum and dividing by the range. This standardisation scales all values to a range between 0 and 1. Grey bars indicate missing data for a given species. Silhouettes represent major taxonomic groups (sourced from www.phylopic.org, public domain).

**Fig. 3:**
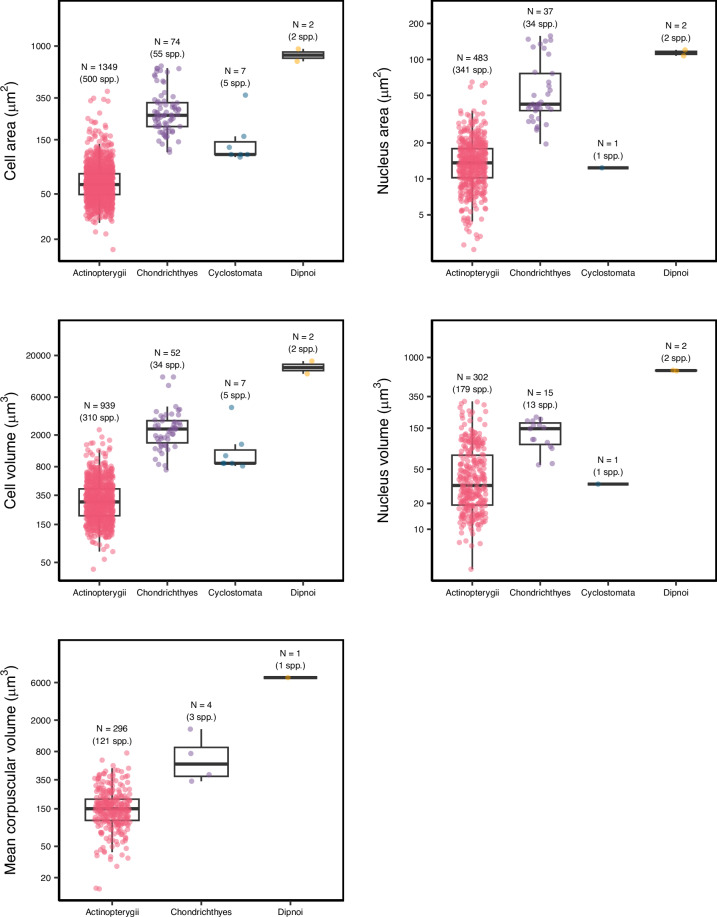
Cell size of erythrocytes among major lineages of fishes: (**A**) cell area (μm^2^), (**B**) nucleus area (μm^2^), (**C**) cell volume (μm3^3^), (**D**) nucleus volume (μm³), and (**E**) mean corpuscular volume (μm³). The number of species (spp.) and records (N) measured for each of the variables is indicated above each box. No data were available on mean corpuscular volume for Cyclostomata. Boxes show median (horizontal line) ± 1.5 times the interquartile range (whiskers). Dots represent observations for each trait.

ErythroCite encompasses over 1,700 records derived from 186 references. After applying the steps of taxonomic harmonization, the final number of unique species included in our database was 660, of which 629 were included in the OTL phylogeny (Fig. 2). In terms of taxonomic diversity, 90.2% were grouped within Actinopterygii (595 species of bony fishes), 8.6% species in Chondrichthyes (57 species of cartilaginous fishes), 0.75% species in Cyclostomata (5 species of jawless fishes) and 0.45% of the species in Dipnoi (3 species of lungfishes) (Fig. 3). To our knowledge, we have compiled the most comprehensive database of erythrocyte (red blood cell) sizes in fish species to date. We anticipate that this database will significantly contribute to understanding the factors influencing cell size variation among fishes and serve as a valuable resource for future research in macroecology, macrophysiology, comparative physiology, and evolutionary biology. However, despite its extensive coverage, our database reveals geographic and taxonomic biases, as well as a lack of reported information in biological metadata. In an ideal scenario, all species included in the current version of ErythroCite would have information on the five traits of interest (Figs. 2, 3), including those from which these traits are derived, such as cellular and nuclear lengths and widths. To address this issue, we foresee the use of phylogenetic imputation methods to fill gaps and to enhance the comprehensiveness of the database^254,255^. This approach could significantly augment the utility of ErythroCite. Specifically, ErythroCite is expected to facilitate research in two key areas: first, by investigating metabolic theories such as the optimal cell size theory^9,12,13,18,256–259^ and hypotheses related to the development of the cardiovascular system in fishes^260^; and second, by examining how external factors such as environmental temperature influence variations in fish cell sizes, particularly the observation that species with larger cells tend to inhabit colder regions like the polar areas^261,262^. These efforts will help identify global-scale variations in cell size by uncovering their underlying causes and analysing their effects. By integrating available metadata, we aim to enhance our understanding of the ecological and evolutionary implications of erythrocyte size diversity in fishes.

## Technical Validation

To validate the entries in the ErythroCite database, we employed various approaches. FPLeiva double-checked all entries resulting from English-language searches. In addition, MShokri and FPLeiva reviewed a subset of studies representing 38% of the total records. No errors were identified during this stage of verification. We established a procedure to examine inconsistencies in our database, methodically detecting, assessing, and rectifying potential deviations in cell size measurements, and also covering both discrete and other continuous variables. For this purpose, we adopted some of the data verification steps outlined by Pottier *et al*.^263^.

We created frequency distribution plots for traits associated with any measure of cell size (cell area, cell volume, nuclear area, nuclear volume, mean corpuscular volume (MCV), cell length, cell width, nuclear length, and nuclear width) to check for outliers. For values in the distribution tails, we conducted checks not only for data entry errors but also for original calculation. Identified errors were corrected in the database. For MCV, we applied the standard formula and verified whether the resulting values closely matched those indicated in the papers. In cases of discrepancy, we considered this calculation as the corrected value, which often proved quite similar and suggested typographical errors in the original article. All these steps and potential corrections were implemented before the release of ErythroCite.

All cellular and nuclear area measurements were expressed in µm², while cell volume, nuclear volume, and MCV were expressed in µm³. Both cell and nucleus length and width were measured in µm.

The majority of entries in our database were derived from tables, primarily aggregated as means at the species, sex, or geographical location level. Alongside the mean values, we documented the corresponding sample sizes, the number of specimens analysed, and the associated error of the mean, which can be valuable for meta-analytic approaches. Where possible, all errors associated with each estimate were converted to standard deviation.

## Data Availability

The data are archived in https://github.com/felixpleiva/ErythroCite and on Zenodo^253^ under the 10.5281/zenodo.18543381.

## Code availability

The code used for generating the figures and tables for this study is available at https://felixpleiva.github.io/ErythroCite/.

## References

[1] Brown, J. H. & West, G. B. *Scaling in Biology*. (Oxford University Press, USA, 2000).

[2] Mohandas, N. & Gallagher, P. G. Red cell membrane: past, present, and future. *Blood, The Journal of the American Society of Hematology* **112**, 3939–3948 (2008). 10.1182/blood-2008-07-161166 PMC2582001 18988878

[3] van Leeuwenhoek, A. Other microscopical observations made by the same, about the texture of the blood, the sap of some plants, the figures of sugar and salt, and the probable cause of the difference of their tastes. *Philosophical Transactions of the Royal Society of London* **10**, 380–385 (1675).

[4] Gulliver, G. Observations on the Sizes and Shapes of the Red Corpuscles of the Blood of Vertebrates, with drawings of them to a unifom scale, and extended and revised tables of measurements. in *Proceedings of the Zoological Society* 474–495 (1875).

[5] Fänge, R. Blood cells, haemopoiesis and lymphomyeloid tissues in fish. *Fish & Shellfish Immunology* **4**, 405–411 (1994).

[6] Farrell, A. P. *Encyclopedia of Fish Physiology: From Genome to Environment*. (Academic Press, 2011).

[7] Boutilier, R. G. & Ferguson, R. A. Nucleated red cell function: metabolism and pH regulation. *Can. J. Zool.* **67**, 2986–2993 (1989).

[8] Wells, R. M. Blood-gas transport and hemoglobin function: Adaptations for functional and environmental hypoxia. in *Fish physiology***vol. 27** 255–299 (Elsevier, 2009).

[9] Szarski, H. Cell size and the concept of wasteful and frugal evolutionary strategies. *Journal of Theoretical Biology* **105**, 201–209 (1983). 6656279 10.1016/s0022-5193(83)80002-2

[10] Szarski, H. Changes in the amount of DNA in cell nuclei during vertebrate evolution. *Nature* **226**, 651–652 (1970). 5444929 10.1038/226651a0

[11] Witeska, M. Erythrocytes in teleost fishes: a review. *Zoology and Ecology* **23**, 275–281 (2013).

[12] Leiva, F. P., Boerrigter, J. G. & Verberk, W. C. E. P. The role of cell size in shaping responses to oxygen and temperature in fruit flies. *Functional Ecology* **37**, 1269–1279 (2023).

[13] Leiva, F. P., Santos, M., Rezende, E. L. & Verberk, W. C. E. P. Intraspecific variation of heat tolerance in a model ectotherm: The role of oxygen, cell size and body size. *Functional Ecology* **38**, 439–448 (2024).

[14] Privalova, V., Sobczyk, Ł., Szlachcic, E., Labecka, A. M. & Czarnoleski, M. Heat tolerance in *Drosophila melanogaster* is influenced by oxygen conditions and mutations in cell size control pathways. *Phil. Trans. R. Soc. B* **379**, 20220490 (2024). 38186282 10.1098/rstb.2022.0490 PMC10772611

[15] Verspagen, N., Leiva, F. P., Janssen, I. & Verberk, W. C. E. P. Effects of developmental plasticity on heat tolerance may be mediated by changes in cell size in *Drosophila melanogaster*. *Insect Science* **27**, 1244–1256 (2020). 31829515 10.1111/1744-7917.12742 PMC7687148

[16] Szlachcic, E., Labecka, A. M., Privalova, V., Sikorska, A. & Czarnoleski, M. Systemic orchestration of cell size throughout the body: influence of sex and rapamycin exposure in *Drosophila melanogaster*. *Biol. Lett.* **19**, 20220611 (2023). 36946132 10.1098/rsbl.2022.0611 PMC10031402

[17] Kozłowski, J., Czarnoleski, M., François-Krassowska, A., Maciak, S. & Pis, T. Cell size is positively correlated between different tissues in passerine birds and amphibians, but not necessarily in mammals. *Biology Letters*, (2010). rsbl20100288 (. 10.1098/rsbl.2010.0288 PMC3001361 20462886

[18] Czarnoleski, M. *et al*. Concerted evolution of body mass and cell size: similar patterns among species of birds (Galliformes) and mammals (Rodentia). *Biology open* bio-029603 (2018). 10.1242/bio.029603 PMC5936057 29540429

[19] Malerba, M. E. & Marshall, D. J. Larger cells have relatively smaller nuclei across the Tree of Life. *Evolution letters* **5**, 306–314 (2021). 34367657 10.1002/evl3.243 PMC8327945

[20] Froese, R. & Pauly, D. FishBase. World Wide Web electronic publication. www.fishbase.org, (ver. 06/2024). (2024).

[21] Witeska, M., Kondera, E., Ługowska, K. & Bojarski, B. Hematological methods in fish–Not only for beginners. *Aquaculture* **547**, 737498 (2022).

[22] Gregory, T. R. Animal genome size database. http://www.genomesize.com/ (2024).

[23] Nakagawa, S. *et al*. Method Reporting with Initials for Transparency (MeRIT) promotes more granularity and accountability for author contributions. *Nature Communications* **14**, 1788 (2023). 37012240 10.1038/s41467-023-37039-1 PMC10070262

[24] Harzing, A. W. Publish or perish, available from https://harzing.com/resources/publish-or-perish. (2007).

[25] Ouzzani, M., Hammady, H., Fedorowicz, Z. & Elmagarmid, A. Rayyan—a web and mobile app for systematic reviews. *Syst Rev* **5**, 210 (2016). 27919275 10.1186/s13643-016-0384-4 PMC5139140

[26] Janko, K., Eisner, J., Cigler, P. & Tichopád, T. Unifying framework explaining how parental regulatory divergence can drive gene expression in hybrids and allopolyploids. *Nature Communications* **15**, 8714 (2024). 39379366 10.1038/s41467-024-52546-5 PMC11461870

[27] Sheikh, Z. A. & Ahmed, I. Comparative evaluation of two anticoagulants used for the analysis of haematological, biochemical parameters and blood cell morphology of himalayan snow trout, *Schizopyge plagiostomus*. *Tissue and Cell* **67**, 101398 (2020). 32835933 10.1016/j.tice.2020.101398

[28] Abd-ElRaouf, M., Moustafa, M., Badrey, A. & Said, R. Hemato-Serological Findings as Early signals in Nile Tilapia *Oreochromis niloticus* Treated with Benzalkonium Chloride. *Baghdad Science Journal* **20**, 945–956 (2023).

[29] Acar, Ü., Kesbiç, O., Yilmaz, S. & Karabayir, A. Growth performance, haematological and serum biochemical profiles in rainbow trout (*Oncorhynchus mykiss*) fed diets with varying levels of lupin (*Lupinus albus*) meal. *Aquaculture Research* **49**, 2579–2586 (2018).

[30] Acar, Ü. *et al*. Comparative study on haematological and biochemical parameters of two wild sparid fish species. *Cahiers de Biologie Marine* **60**, 51–57 (2019).

[31] Acharya, G. & Mohanty, P. K. Effect of sex on haemocytobiochemical profiling of silver tiger fish (*Datnioides polota* Hamilton, 1822). *Comparative Clinical Pathology* **27**, 1335–1342 (2018).

[32] Acharya, G. & Mohanty, P. K. Comparative cytomorphometry of red blood cells of some fishes. *African Journal of Biological Sciences (South Africa)* **1**, 23–32 (2019).

[33] Agrawal, N. & Mahajan, C. Hematological and hematopoietic studies in pyridoxine deficient fish, *Channa punctatus* Bloch. *Journal of Fish Biology* **22**, 91–103 (1983).

[34] Ahmed, I. & Sheikh, Z. A. Comparative study of hematological parameters of snow trout *Schizopyge plagiostomus* and *Schizopyge niger* inhabiting two different habitats. *European Zoological Journal* **87**, 12–19 (2020).

[35] Al-Emran, M. *et al*. Alterations in hematological parameters and the structure of peripheral erythrocytes in Nile tilapia (*Oreochromis niloticus*) exposed to profenofos. *Environmental Science and Pollution Research* **29**, 29049–29061 (2022). 34993795 10.1007/s11356-021-17972-8

[36] Alabi, O. Comparative chemical analysis, mutagenicity, and genotoxicity of petroleum refinery wastewater and its contaminated river using prokaryotic and eukaryotic assays. *Protoplasma* **260**, 89–101 (2023). 35467135 10.1007/s00709-022-01763-0

[37] Alaguprathana, M. & Poonkothai, M. Haematological, biochemical, enzymological and histological responses of *Labeo rohita* exposed to methyl orange dye solution treated with *Oedogonium subplagiostomum* AP1. *Environmental Science and Pollution Research* **28**, 17602–17612 (2021). 33400116 10.1007/s11356-020-12208-7

[38] Alak, G., Kotan, R., Uçar, A., Parlak, V. & Atamanalp, M. Pre-probiotic effects of different bacterial species in aquaculture: behavioral, hematological and oxidative stress responses. *Oceanological and Hydrobiological Studies* **51**, 133–142 (2022).

[39] Angeles-Escobar, B., da Silva, S. & Severi, W. Growth, red blood cells, and gill alterations of red pacu *(Piaractus brachypomus)* fingerlings by chronic exposure to different total suspended solids in biofloc. *Journal of the World Aquaculture Society* **53**, 652–668 (2022).

[40] Arnaudov, A., Velcheva, I. & Tomova, E. Influence of copper and zinc on the erythrocyte-metric parameters of *Carassius gibelio* (Pisces, Cyprinidae). *Bulgarian Journal of Agricultural Science* **14**, 557–563 (2008).

[41] Atamanalp, M. *et al*. The Alterations in the Hematological Parameters of Rainbow Trout, *Oncorhynchus mykiss*, Exposed to Cobalt Chloride. *Kafkas Universitesi Veteriner Fakultesi Dergisi* **17**, S73–S76 (2011).

[42] Atamanalp, M., Kocaman, E., Ucar, A. & Alak, G. The Alterations in the Hematological Parameters of Brown Trout *Salmo trutta fario*, Exposed to Cobalt Chloride. *Journal of Animal and Veterinary Advances* **9**, 2167–2170 (2010).

[43] Atencio-García, V., Genes López, F., Madariaga Mendoza, D. & Pardo Carrasco, S. Hematología y química sanguínea de juveniles de rubio (*Salminus affinis* Pisces: Characidae) del río Sinú. *Acta Biológica Colombiana* **12**, 27–40 (2007).

[44] Bachmann, K. & Cowden, R. Specific DNA amounts and nuclear size in fish hepatocytes and erythrocytes. *Transactions of the American Microscopical Society* **86**, 463–471 (1967). 4169469

[45] Baghizadeh, E. & Khara, H. Variability in hematology and plasma indices of common carp *Cyprinus carpio*, associated with age, sex and hormonal treatment. *Iranian Journal of Fisheries Sciences* **14**, 99–111 (2015).

[46] Baldisserotto, B. *et al*. Ion fluxes and hematological parameters of two teleosts from the Rio Negro, Amazon, exposed to hypoxia. *Brazilian Journal of Biology* **68**, 571–575 (2008). 10.1590/s1519-69842008000300015 18833479

[47] Bani, A. & Vayghan, A. Temporal variations in haematological and biochemical indices of the Caspian kutum, *Rutilus frisii* Kutum. *Ichthyological Research* **58**, 126–133 (2011).

[48] Barham, W., Smit, G. & Schoonbee, H. The hematological assessment of bacterial-infection in rainbow-trout, *Salmo gairdneri* Richardson. *Journal of Fish Biology* **17**, 275–281 (1980).

[49] Berillis, P. *et al*. Improving aeration for efficient oxygenation in sea bass sea cages. Blood, brain and gill histology. *Open Life Sciences* **11**, 270–279 (2016).

[50] Bianchi, M. *et al*. The hematological profile of farmed *Sorubitn lima*: reference intervals, cell morphology and cytochemistry. *Veterinarski Arhiv* **84**, 677–690 (2014).

[51] Boggs, T., Friedman, J. & Gross, J. Alterations to cavefish red blood cells provide evidence of adaptation to reduced subterranean oxygen. *Scientific Reports* **12**, (2022). 10.1038/s41598-022-07619-0 PMC8904627 35260642

[52] Brill, R., Bushnell, P., Schroff, S., Seifert, R. & Galvin, M. Effects of anaerobic exercise accompanying catch-and-release fishing on blood-oxygen affinity of the sandbar shark (*Carcharhinus plumbeus*, Nardo). *Journal of Experimental Marine Biology and Ecology* **354**, 132–143 (2008).

[53] Bytyutskyy, D., Kholodnyy, V. & Flajshans, M. 3-D structure, volume, and DNA content of erythrocyte nuclei of polyploid fish. *Cell Biology International* **38**, 708–715 (2014). 24446105 10.1002/cbin.10247

[54] de Camargo, D. J. Suplementação mineral e vitamínica em dietas para alevinos de tilápia do Nilo. (Universidade Estadual do Oeste do Paraná, Toledo, 2013).

[55] Cazenave, J., Wunderlin, D., Hued, A. & Bistoni, M. Haematological parameters in a neotropical fish, *Corydoras paleatus* (Jenyns, 1842) (Pisces, Callichthyidae), captured from pristine and polluted water. *Hydrobiologia* **537**, 25–33 (2005).

[56] Çelik, E., Kaya, H., Yilmaz, S. & Çakici, H. Effect of Water Temperature, Salinity, Season, Reproduction, Sex, Size, and Age on Hematological Parameters of Horse Mackerel (*Trachurus trachurus*). *Kafkas Universitesi Veteriner Fakultesi Dergisi* **18**, 551–558 (2012).

[57] Chaudhary, A., Javaid, K. & Bughio, E. Toxic effects of chromium chloride on hematology and histopathology of major carp *(Labeo rohita)*. *Egyptian Journal of Aquatic Research* **49**, 291–296 (2023).

[58] Chen, H., Yuan, G., Su, J. & Liu, X. Hematological analysis of *Ctenopharyngodon idella*, *Megalobrama amblycephala* and *Pelteobagrus fulvidraco*: Morphology, ultrastructure, cytochemistry and quantification of peripheral blood cells. *Fish and Shellfish Immunology* **90**, 376–384 (2019). 31048039 10.1016/j.fsi.2019.04.044

[59] Cieplinski, M. *et al*. The effect of dipotassium EDTA and lithium heparin on hematologic values of farmed brown trout *Salmo trutta* (L.) spawners. *Aquaculture International* **27**, 79–87 (2019).

[60] Cleland, J. B. & Johnston, T. H. Relative dimensions of the red blood cells of vertebrates, especially of birds. *Emu-Austral Ornithology* **11**, 188–197 (1912).

[61] Conroy, D. A. & Rodriguez, J. L. Erythrocyte Measurements of Some Argentine Fishes. *The Progressive Fish-Culturist* **28**, 46–46 (1966).

[62] Correa Negrete, J. C., Garrido Correa, A. A., Prieto Guevara, M. J., Atencio García, V. J. & Pardo Carrasco, S. C. Caracterización de células sanguíneas y parámetros hematológicos en blanquillo *Sorubim cuspicaudus*. *Zootecnia tropical* **27**, 393–405 (2009).

[63] Daneshvar, E., Ardestani, M., Dorafshan, S. & Martins, M. Hematological parameters of Iranian cichlid *Iranocichla hormuzensis* - Coad, 1982 (Perciformes) in Mehran River. *Anais da Academia Brasileira de Ciencias* **84**, 943–949 (2012). 22935922 10.1590/s0001-37652012005000054

[64] Das, S. *et al*. Study on impacts of direct supplementation of choline into semi-intensive fish culture system based on haematopoietic alterations. *Environmental and Sustainability Indicators* **9**, (2021).

[65] Dekic, R. *et al*. Hematological characteristics of *Delminichthys ghetaldii* (Steindachner 1882) inhabiting the Karst region of Eastern Herzegovina. *Archives of Biological Sciences* **66**, 1423–1430 (2014).

[66] Dhillon, S. & Gupta, A. A clinical approach to study the pollutants intoxication in a fresh-water teleost *Clarias batrachus*. *Water Air and Soil Pollution* **20**, 63–68 (1983).

[67] Dorafshan, S., Kalbassi, M., Pourkazemi, M., Amiri, B. & Karimi, S. Effects of triploidy on the Caspian salmon *Salmo trutta caspius* haematology. *Fish Physiology and Biochemistry* **34**, 195–200 (2008). 18665456 10.1007/s10695-007-9176-z

[68] Dorafshan, S., Kalbassi, M. R., Karimi, S. S. & Rahimi, K. Study of Some Haematological Indices of Diploid and Triploid Rainbow Trout, *Oncorhynchus mykiss*. *Yakhteh Medical Journal* **11**, (2010).

[69] Elahee, K. B. & Bhagwant, S. Hematological and gill histopathological parameters of three tropical fish species from a polluted lagoon on the west coast of Mauritius. *Ecotoxicology and Environmental Safety* **68**, 361–371 (2007). 16879869 10.1016/j.ecoenv.2006.06.003

[70] Emiroglu, Ö., Uyanoglu, M. & Baskurt, S. Comparison of the Erythrocyte Sizes of *Carassius gibelio* and C*arassius carassius* Species Living Together in Akgol (Adapazari/Turkey). *Asian Journal of Animal and Veterinary Advances* **7**, 876–883 (2012).

[71] Evdokimov, E. & Flerova, E. Features of Erythropoiesis of the Mesonephros and Peripheral Blood in *Polypterus senegalus* (Polypteridae). *Journal of Ichthyology* **62**, 1521–1527 (2022).

[72] Fagbenro, O., Adedire, C., Ayotunde, E. & Faminu, E. Haematological profile, food composition and digestive enzyme assay in the gut of the African bony-tongue fish, *Heterotis* (*Clupisudis*) *niloticus* (Cuvier 1829) (Osteoglossidae). *Tropical Zoology* **13**, 1–9 (2000).

[73] Faggio, C. *et al*. Effect of storage time on haematological parameters in mullet, *Mugil cephalus*. *Cell Biochemistry and Function* **31**, 412–416 (2013). 23097308 10.1002/cbf.2915

[74] Fang, J. *et al*. Morphological and Cytochemical Studies of Peripheral Blood Cells of *Schizothorax prenanti*. *Anatomia Histologia Embryologia* **43**, 386–394 (2014). 24117489 10.1111/ahe.12089

[75] Fazio, F. *et al*. Biochemical and hematological parameters in European sea bass (*Dicentrarchus labrax* Linnaeus, 1758) and Gilthead sea bream (*Sparus aurata* Linnaeus, 1758) in relation to temperature. *Veterinarski Arhiv* **88**, 397–411 (2018).

[76] Fazio, F., Ferrantelli, V., Saoca, C., Giangrosso, G. & Piccione, G. Stability of haematological parameters in stored blood samples of rainbow trout *Oncorhynchus mykiss* (Walbaum, 1792). *Veterinarni Medicina* **62**, 401–405 (2017).

[77] Fazio, F. *et al*. Individual variability of blood parameters in striped bass *Morone saxatilis*: possible differences related to weight and length. *Aquaculture International* **28**, 1665–1673 (2020).

[78] Filipiak, M., Tylko, G. & Kilarski, W. Flow cytometric determination of genome size in European sunbleak *Leucaspius delineatus* (Heckel, 1843). *Fish Physiology and Biochemistry* **38**, 355–362 (2012). 21614549 10.1007/s10695-011-9512-1 PMC3309147

[79] Flajshans, M., Psenicka, M., Rodina, M. & Tesitel, J. Image cytometric measurements of diploid, triploid and tetraploid fish erythrocytes in blood smears reflect the true dimensions of live cells. *Cell Biology International* **35**, 67–71 (2011). 20812918 10.1042/CBI20100198

[80] Fris, J., Deki, R., Ivanc, A. & Kukavica, B. Superoxide dismutase and oxygen transport mechanism in endemic fish *Delminichthys ghetaldii* (Steindachner, 1882) under mild hypoxia. *Indian Journal of Experimental Biology* **62**, 410–415 (2024).

[81] Fukushima, H., Bailone, R., Weiss, L., Martins, M. & Zaniboni, E. Triploidy in the hematology of jundia juveniles (Siluriformes: Heptapteridae). *Brazilian Journal of Biology* **72**, 147–151 (2012). 10.1590/s1519-69842012000100017 22437395

[82] Galeano, N. A., Prat, M. I., Guagliardo, S. E., Schwerdt, C. B. & Tanzola, R. D. Características hematológicas de *Porichthys porosissimus* (Pisces: batrachoidiformes) en el estuario de Bahía Blanca, Argentina. *Analecta Veterinaria* **30**, 5–11 (2010).

[83] Galina, M. The difference in brown trout (*Salmo trutta* L) blood composition from acidic and limed sites of two rivers in western Norway. *Water Air and Soil Pollution* **96**, 203–210 (1997).

[84] Garcia-Abiado, M., Dabrowski, K., Christensen, J., Czesny, S. & Bajer, P. Use of erythrocyte measurements to identify triploid saugeyes. *North American Journal of Aquaculture* **61**, 319–325 (1999).

[85] Gayatri, A. & Prafulla, M. The morphometrical characterisation of normal blood cells of two airbreathing fishes, *Clarias batrachus* and *Anabas testudineus*. *International Research Journal of Biological Sciences* **3**, 37–41 (2014).

[86] Habibi, S. *et al*. Comparative Analysis of Hematological Parameters of some Farmed and Wild Fish Species. *Pakistan Journal of Zoology* **54**, 591–598 (2022).

[87] Hamed, M. *et al*. Exposure to pyrogallol impacts the hemato-biochemical endpoints in catfish (*Clarias gariepinus*). *Environmental Pollution* **333**, (2023). 10.1016/j.envpol.2023.122074 37331582

[88] Han, G., Yao, H., Qiang, L., Chen, X. & Gao, Y. Comparative study of peripheral blood cells in two marine fishes (*Synechogobius hasta* and *Sebastes schlegelii*): Morphological and cytochemical characterization. *Tissue and Cell* **73**, (2021). 10.1016/j.tice.2021.101633 34534744

[89] Hardie, D. C. & Hebert, P. D. N. The nucleotypic effects of cellular DNA content in cartilaginous and ray-finned fishes. *Genome* **46**, 683–706 (2003). 12897876 10.1139/g03-040

[90] Hardig, J. & Hoglund, L. On accuracy in estimating fish blood variables. *Comparative Biochemistry and Physiology A* **75**, 35–40 (1983).

[91] Hardig, J. & Hoglund, L. Seasonal-variation in blood components of reared Baltic salmon, *Salmo salar* L. *Journal of Fish Biology* **24**, 565–579 (1984).

[92] Hartman, F. & Lessler, M. Erythrocyte measurements in fishes, amphibia, and reptiles. *Biological Bulletin* **126**, 83–88 (1964).

[93] Hasan, Z., Ghayyur, S., Hassan, Z. & Rafique, S. Histomorphometric and Hematological Profile of Grass Carp (*Ctenopharyngodon idella*) during Acute Endosulfan Toxicity. *Pakistan Veterinary Journal* **35**, 23–27 (2015).

[94] Hedayati, A. & Tarkhani, R. Hematological and gill histopathological changes in iridescent shark, *Pangasius hypophthalmus* (Sauvage, 1878) exposed to sublethal diazinon and deltamethrin concentrations. *Fish Physiology and Biochemistry* **40**, 715–720 (2014). 24126937 10.1007/s10695-013-9878-3

[95] Hemalatha, D., Muthukumar, A., Rangasamy, B., Nataraj, B. & Ramesh, M. Impact of sublethal concentration of a fungicide propiconazole on certain health biomarkers of Indian major carp *Labeo rohita*. *Biocatalysis and Agricultural Biotechnology* **8**, 321–327 (2016).

[96] Hoffmann, R. & Lommel, R. Effects of repeated blood-sampling on some blood parameters in freshwater fish. *Journal of Fish Biology* **24**, 245–251 (1984).

[97] Hoseini, S. & Ghelichpour, M. Efficacy of clove solution on blood sampling and hematological study in Beluga, *Huso huso* (L.). *Fish Physiology and Biochemistry* **38**, 493–498 (2012). 21691726 10.1007/s10695-011-9529-5

[98] Hosseini, S. & Khajepour, F. Effect of partial replacement of dietary fish meal with soybean meal on some hematological and serum biochemical parameters of juvenile beluga, *Huso huso*. *Iranian Journal of Fisheries Sciences* **12**, 348–356 (2013).

[99] Hughes, G., Kikuchi, Y. & Barrington, J. Physiological salines and the mechanical-properties of trout red-blood-cells. *Journal of Fish Biology* **29**, 393–402 (1986).

[100] Hughes, G., Kikuchi, Y. & Watari, H. A study of the deformability of red-blood-cells of a teleost fish, the yellowtail (*Seriola quinqueradiata*), and a comparison with human-erythrocytes. *Journal of Evolutionary Biology* **96**, 209–220 (1982). 10.1242/jeb.96.1.209 7077219

[101] Ihut, A. *et al*. Seasonal variation of blood biomarkers in huchen, *Hucho hucho* (Actinopterygii: Salmoniformes: Salmonidae) reared in captivity. *Acta Ichthyologica et Piscatoria* **50**, 381–390 (2020).

[102] Islam, S. *et al*. Acute effects of chromium on hemato-biochemical parameters and morphology of erythrocytes in striped catfish *Pangasianodon hypophthalmus*. *Toxicology Reports* **7**, 664–670 (2020). 32489906 10.1016/j.toxrep.2020.04.016 PMC7260616

[103] Jaffer, N., Rabee, A. & Al-Chalabi, S. Biochemical and hematological parameters and histological alterations in fish *Cyprinus carpio* L. as biomarkers for water pollution with chlorpyrifos. *Human and Ecological Risk Assessment* **23**, 605–616 (2017).

[104] Jagoe, C. & Welter, D. Quantitative comparisons of the morphology and ultrastructure of erythrocyte nuclei from seven freshwater fish species. *Canadian Journal of Zoology-Revue Canadienne De Zoologie* **73**, 1951–1959 (1995).

[105] Jayaprasad, P. *et al*. Identification of Diploid and Triploid Red Tilapia by Using Erythrocyte Indices. *Caryologia* **64**, 485–492 (2011).

[106] Jeamah, A. *et al*. Hematological Evaluation of Three Common Teleosts in Relation to The Environmental Changes from Trang Province, Thailand. *Tropical Life Sciences Research* **34**, 113–127 (2023). 37860093 10.21315/tlsr2023.34.3.6 PMC10583840

[107] Jensen, F. *et al*. Anion exchange in the giant erythrocytes of African lungfish. *Journal of Fish Biology* **62**, 1044–1052 (2003).

[108] John, P. Alteration of certain blood parameters of freshwater teleost *Mystus vittatus* after chronic exposure to Metasystox and Sevin. *Fish Physiology and Biochemistry* **33**, 15–20 (2007).

[109] Jung, S., Sim, D., Park, M., Jo, Q. & Kim, Y. Effects of formalin on haematological and blood chemistry in olive flounder, *Paralichthys olivaceus* (Temminck et Schlegel). *Aquaculture Research* **34**, 1269–1275 (2003).

[110] Kavadias, S., Castritsi-Catharios, J., Dessypris, A. & Miliou, H. Seasonal variation in steroid hormones and blood parameters in cage-farmed European sea bass (*Dicentrarchus labrax* L.). *Journal of Applied Ichthyology* **20**, 58–63 (2004).

[111] Khan, N. *et al*. Effects of Sub-Lethal Concentration of Cypermethrin on Histopathological and Hematological Profile of Rohu (*Labeo rohita*) during Acute Toxicity. *International Journal of Agriculture and Biology* **20**, 601–608 (2018).

[112] Khatun, H., Mostakim, G. & Islam, S. Acute responses of spotted snakehead (*Channa punctata*) to salinity stress: A study of a freshwater fish to salinity challenges during intrusion of saline water. *Iranian Journal of Fisheries Sciences* **19**, 2673–2687 (2020).

[113] Kisch, B. Hemoglobin content, size and amount of erythrocytes in fishes. *Experimental medicine and surgery* **7**, 118–133 (1949). 18151280

[114] Kisch, B. Observations on the haematology of fishes and birds. *Experimental medicine and surgery* **7**, 318–326 (1949). 15396127

[115] Kisch, B. Erythrocytes in fishes. *Experimental medicine and surgery* **9**, 125–137 (1951). 14813273

[116] Knoph, M. & Thorud, K. Toxicity of ammonia to Atlantic salmon (*Salmo salar* L) in seawater - Effects on plasma osmolality, ion, ammonia, urea and glucose levels and hematologic parameters. *Comparative Biochemistry and Physiology A* **113**, 375–381 (1996).

[117] Kori-Siakpere, O., Ake, J. & Idoge, E. Haematological characteristics of the African snakehead, *Parachanna obscura*. *African Journal of Biotechnology* **4**, 527–530 (2005).

[118] Kumar, M. V. Morphometric studies of blood cells in *Cyprinus carpio*, *Ctenopharyngodan idella* and *Hypophthalmichthys molitrix* cultured fish in west Godavari region of Andhra Pradesh. *International Journal of Fisheries and Aquatic Studies* **4**, 489–493 (2016).

[119] Kumar, S., Raman, R., Prasad, K., Srivastava, P. & Rajendran, K. Effects on haematological and serum biochemical parameters of *Pangasianodon hypophthalmus* to an experimental infection of *Thaparocleidus sp* (Monogenea: dactylogyridae). *Experimental Parasitology* **188**, 1–7 (2018). 29501694 10.1016/j.exppara.2018.02.007

[120] Kumar, S. *et al*. Effect of orally administered azadirachtin on non-specific immune parameters of goldfish *Carassius auratus* (Linn. 1758) and resistance against *Aeromonas hydrophila*. *Fish and Shellfish Immunology* **34**, 564–573 (2013). 23261511 10.1016/j.fsi.2012.11.038

[121] Lahnsteiner, F. Erythrocyte morphometry in teleost fish-Species-specific, inter-individual and environmental-related differences. *Acta Zoologica* **102**, 237–249 (2021).

[122] Lay, P. & Baldwin, J. What determines the size of teleost erythrocytes? Correlations with oxygen transport and nuclear volume. *Fish Physiology and Biochemistry* **20**, 31–35 (1999).

[123] Lewis, J. H. *Comparative Hemostasis in Vertebrates*. (Springer Science & Business Media, 2013).

[124] Luo, Y. *et al*. Intraspecific metabolic scaling exponent depends on red blood cell size in fishes. *Journal of Experimental Biology* **218**, 1496–1503 (2015). 25795736 10.1242/jeb.117739

[125] Lutnicka, H. *et al*. Hematological Alterations as a Response to Exposure to Selected Fungicides in Common Carp (*Cyprinus carpio* L.). *Folia Biologica-Krakow* **64**, 235–244 (2016). 10.3409/fb64_4.235 29809362

[126] Machado, M. *et al*. Acute hyperoxia induces systemic responses with no major changes in peripheral tissues in the Senegalese sole (*Solea senegalensis* Kaup, 1858). *Fish and Shellfish Immunology* **74**, 260–267 (2018). 29325709 10.1016/j.fsi.2018.01.008

[127] Martins, B., Franco-Belussi, L., Siqueira, M., Fernandes, C. & Provete, D. The evolution of red blood cell shape in fishes. *Journal of Evolutionary Biology* **34**, 537–548 (2021). 33484056 10.1111/jeb.13757

[128] Martins, M. L., Tavares-Dias, M., Fujimoto, R., Onaka, E. & Nomura, D. Haemalogical alterations of *Leporinus macrocephalus* (Osteichtyes: Anostomidae) naturally infected by *Goezia leporini* (Nematoda: Anisakidae) in fish pont. *Arquivo Brasileiro de Medicina Veterinaria e Zootecnia* **56**, 640–646 (2004).

[129] Mavares, R. & Perez, J. Blood adaptations to marine and fresh-water environments in fish of the family Sciaenidae (Perciformes). *Journal of Fish Biology* **25**, 657–663 (1984).

[130] Mekkawy, I., Mahmoud, U. & Sayed, A. Effects of 4-nonylphenol on blood cells of the African catfish *Clarias gariepinus* (Burchell, 1822). *Tissue and Cell* **43**, 223–229 (2011). 21501852 10.1016/j.tice.2011.03.006

[131] Motlagh, S. P., Zarejabad, A. M., Nasrabadi, R. G., Ahmadifar, E. & Molaee, M. Haematology, morphology and blood cells characteristics of male and female Siamese fighting fish (*Betta splendens*). *Comparative Clinical Pathology* **21**, 15–21 (2012).

[132] Murad, A. & Houston, A. Haemoglobin isomorph abundances in splenectomized rainbow trout, *Oncorhynchus mykiss* (Walbaum). *Journal of Fish Biology* **38**, 641–651 (1991).

[133] Murray, S. & Burton, C. Effects of density on goldfish blood .2. Cell morphology. *Comparative Biochemistry and Physiology A-Molecular & Integrative Physiology* **62**, 559–562 (1979).

[134] Muthukumaravel, K. *et al*. Impact of sublethal phenol in freshwater fish *Labeo rohita* on biochemical and haematological parameters. *Environmental Monitoring and Assessment* **195**, (2023). 10.1007/s10661-022-10554-2 36269455

[135] Nabi, N., Ahmed, I. & Wani, G. Hematological and serum biochemical reference intervals of rainbow trout, *Oncorhynchus mykiss* cultured in Himalayan aquaculture: Morphology, morphometrics and quantification of peripheral blood cells. *Saudi Journal of Biological Sciences* **29**, 2942–2957 (2022). 35531244 10.1016/j.sjbs.2022.01.019 PMC9073141

[136] Naeem, Z., Zuberi, A., Ali, M., Naeem, A. & Naeem, M. Toxic effects of ammonia exposure on growth and hematological response of *Clarias batrachus* (Linneaeus, 1758). *Applied Ecology and Environmental Research* **21**, 5055–5067 (2023).

[137] Natarajan, G. Effect of sublethal concentration of metasystox on selected oxidative-enzymes, tissue respiration, and hematology of the fresh-water air-breathing fish, *Channa striatus* (Bleeker). *Pesticide Biochemistry and Physiology* **21**, 194–198 (1984).

[138] Neale, N., Honn, K. & Chavin, W. Hematological responses to thermal acclimation in a cold water squaliform (*Heterodontus francisci* Girard). *Journal of Comparative Physiology* **115**, 215–222 (1977).

[139] Nespolo, R. & Rosenmann, M. Intraspecific allometry of haematological parameters in *Basilichthys australis*. *Journal of Fish Biology* **60**, 1358–1362 (2002).

[140] Noleto, R. *et al*. Genome Size Evaluation in Tetraodontiform Fishes from the Neotropical Region. *Marine Biotechnology* **11**, 680–685 (2009). 19590923 10.1007/s10126-009-9215-0

[141] Olena, F., Natalia, Y. & Tetyana, S. The accumulation of heavy metals and cytometric characteristics features of red blood cells in different ages of carp fish from Zaporozhian Reservoir. *International Letters of Natural Sciences* **53**, 72–79 (2016).

[142] Olusola, S. & Nwokike, C. Effects of dietary leaves extracts of bitter (*Vernonia amygdalina*) and pawpaw (*Carica papaya*) on the growth, feed conversion efficiency and disease resistance on juveniles *Clarias gariepinus*. *Aquaculture Research* **49**, 1858–1865 (2018).

[143] Pages, T., Gomez, E., Suner, O., Viscor, G. & Tort, L. Effects of daily management stress on hematology and blood rheology of the gilthead seabream. *Journal of Fish Biology* **46**, 775–786 (1995).

[144] Pandey, K. & Pandey, A. Hematology of a cat fish *Rita rita* (Ham). *Proceedings of the Indian Academy of Sciences Section B* **85**, 369–377 (1977).

[145] Panis, C. & Souza, M. Methods of measuring volume changes in erythrocytes under hypoosmotic stress - A comparison. *Analytical and Quantitative Cytology and Histology* **27**, 95–100 (2005). 15913202

[146] Park, I., Hur, J. & Choi, J. Hematological Responses, Survival, and Respiratory Exchange in the Olive Flounder, *Paralichthys olivaceus*, during Starvation. *Asian-Australasian Journal of Animal Sciences* **25**, 1276–1284 (2012). 25049691 10.5713/ajas.2012.12128 PMC4092947

[147] Parrino, V. *et al*. Comparative study of haematology of two teleost fish (*Mugil cephalus* and *Carassius auratus*) from different environments and feeding habits. *European Zoological Journal* **85**, 194–200 (2018).

[148] Pedlar, R., Ptashynski, M., Evans, R. & Klaverkamp, J. Toxicological effects of dietary arsenic exposure in lake whitefish (*Coregonus clupeaformis*). *Aquatic Toxicology* **57**, 167–189 (2002). 11891005 10.1016/s0166-445x(01)00198-9

[149] Petrillo, T. R. Levamisol e dexametasona na inflamação crônica por corpo estranho em Pacu (*Piaractus mesopotamicus*). (Universidade Estadual Paulista (Unesp), 2012).

[150] Potter, I. C., Percy, L. R., Barber, D. L. & Macey, D. J. The morphology, development and physiology of blood cells. in *The biology of lampreys* 233–292 (Academic Press, 1982).

[151] Priya, K., Ramesh, M., Saravanan, M. & Ponpandian, N. Ecological risk assessment of silicon dioxide nanoparticles in a freshwater fish *Labeo rohita*: Hematology, ionoregulation and gill Na^+^/K^+^ ATPase activity. *Ecotoxicology and Environmental Safety* **120**, 295–302 (2015). 26094035 10.1016/j.ecoenv.2015.05.032

[152] Pund, R. P. Anwendung hämatologischer Untersuchungsmethoden für Fischblut und Beeinflussung des Blutbildes von Bachforellen (*Salmo trutta f. fario*) durch Haltungs-und Umwelteinflüsse sowie endogene Faktoren. (Freie Universität Berlin, Berlin, 1998).

[153] Qiu, Y. *et al*. Induction of micronuclei, nuclear anomalies, and dimensional changes in erythrocytes of the rare minnow (*Gobiocypris rarus*) by lanthanum. *Environmental Science and Pollution Research* **27**, 31243–31249 (2020). 32488715 10.1007/s11356-020-09396-7

[154] Rambhaskar, B. & Srinivasa Rao, K. Comparative haematology of ten species of marine fish from Visakhapatnam Coast. *Journal of Fish Biology* **30**, 59–66 (1987).

[155] Ramesh, M., Thilagavathi, T., Rathika, R. & Poopal, R. Antioxidant status, biochemical, and hematological responses in a cultivable fish *Cirrhinus mrigala* exposed to an aquaculture antibiotic Sulfamethazine. *Aquaculture* **491**, 10–19 (2018).

[156] Rehulka, J. & Adamec, V. Red blood cell indices for rainbow trout (*Oncorhynchus mykiss* Walbaum) reared in cage and raceway culture. *Acta Veterinaria Brno* **73**, 105–114 (2004).

[157] Rehulka, J., Minarík, B. & Rehulková, E. Red blood cell indices of rainbow trout *Oncorhynchus mykiss* (Walbaum) in aquaculture. *Aquaculture Research* **35**, 529–546 (2004).

[158] Ribeiro, C. *et al*. Hematological findings in neotropical fish *Hoplias malabaricus* exposed to subchronic and dietary doses of methylmercury, inorganic lead, and tributyltin chloride. *Environmental Research* **101**, 74–80 (2006). 16388797 10.1016/j.envres.2005.11.005

[159] Rios, F., Oba, E., Fernandes, M., Kalinin, A. & Rantin, F. Erythrocyte senescence and haematological changes induced by starvation in the neotropical fish traira, *Hoplias malabaricus* (Characiformes, Erythrinidae). *Comparative Biochemistry and Physiology A-Molecular & Integrative Physiology* **140**, 281–287 (2005). 10.1016/j.cbpb.2004.12.006 15792593

[160] Romestand, B. Etude écophysiologique des parasitoses à Cymothoadiens. *Annales de Parasitologie humaine et comparée* **54**, 423–448 (1979). 533110

[161] Sadler, J., Wells, R., Pankhurst, P. & Pankhurst, N. Blood oxygen transport, rheology and haematological responses to confinement stress in diploid and triploid Atlantic salmon, *Salmo salar*. *Aquaculture* **184**, 349–361 (2000).

[162] Sado, R., Bicudo, A. & Cyrino, J. Hematology of juvenile pacu, *Piaractus mesopotamicus* (Holmberg, 1887) fed graded levels of mannan oligosaccharides (MOS). *Latin American Journal of Aquatic Research* **42**, 30–39 (2014).

[163] Santana-Piñeros, A., Cruz-Quintana, Y., Reyes-Mero, B., González-Solís, D. & Rodríguez-Canul, R. Hematological parameters of the Pacific fat sleeper, *Dormitator latifrons* (Pisces: Eleotridae), under natural and cultured conditions. *Egyptian Journal of Aquatic Research* **50**, 162–167 (2024).

[164] Saoca, C., Arfuso, F., Giannetto, C., Piccione, G. & Fazio, F. Seasonal Biodistribution of Some Trace Elements (Cd, Pb, Cr, Hg) and ‘Blood Biomarkers’ Response in *Mugil cephalus* (Linnaeus, 1758). *Biological Trace Element Research* **201**, 1987–1995 (2023). 35508888 10.1007/s12011-022-03272-w

[165] Saravanan, M., Kumar, K. & Ramesh, M. Haematological and biochemical responses of freshwater teleost fish *Cyprinus carpio* (Actinopterygii: Cypriniformes) during acute and chronic sublethal exposure to lindane. *Pesticide Biochemistry and Physiology* **100**, 206–211 (2011).

[166] Saunders, D. C. Differential blood cell counts of 121 species of marine fishes of Puerto Rico. *Transactions of the American Microscopical Society* 427–449 (1966). 5936509

[167] Saunders, D. C. Elasmobranch blood cells. *Copeia* **1966**, 348–351 (1966).

[168] Sayed, A., Mahmoud, U. & Muhammad, O. Comparative study of two carnivorous fish (*Parupeneus forsskali and Thalassoma klunzingeri*) from the Red Sea: Hemato-biochemical parameters and cellular characterization. *Tissue and Cell* **63**, (2020). 10.1016/j.tice.2019.101316 32223945

[169] Schutt, D., Lehmann, J., Goerlich, R. & Hamers, R. Haematology of swordtail, *Xiphophorus helleri* .1. Blood parameters and light microscopy of blood cells. *Journal of Applied Ichthyology* **13**, 83–89 (1997).

[170] Seol, D. *et al*. Haematological parameters and respiratory function in diploid and triploid Far Eastern catfish, *Silurus asotus*. *Genes and Genomics* **30**, 205–213 (2008).

[171] Serezli, R., Kucukagtas, A. & Kurtoglu, I. Acute toxicity of ammonia and nitrite to angel fish (*Pterophyllum scalare*, Liechtenstein 1823) and the effect of erythrocyte morphology. *Fresenius Environmental Bulletin* **25**, 3119–3124 (2016).

[172] Seriani, R. *et al*. *In vitro* mucus transportability, cytogenotoxicity, and hematological changes as non-destructive physiological biomarkers in fish chronically exposed to metals. *Ecotoxicology and Environmental Safety* **112**, 162–168 (2015). 25463867 10.1016/j.ecoenv.2014.11.003

[173] Sezaki, K., Kobayasi, H., Watabe, S. & Hashimoto, K. Erythrocyte size and polyploidy of cobitid fishes in Japan. *Bulletin Of the Japanese Society of Scientific Fisheries* **51**, 777–781 (1985).

[174] Shah, S. Impairment in the haematological parameters of tench (*Tinca tinca*) infected by *Saprolegnia* spp. *Turkish Journal of Veterinary & Animal Sciences* **34**, 313–318 (2010).

[175] Sheikh, Z. A. & Ahmed, I. Comparative evaluation of two anticoagulants used for the analysis of haematological, biochemical parameters and blood cell morphology of himalayan snow trout, *Schizopyge plagiostomus*. *Tissue and Cell* **67**, (2020). 10.1016/j.tice.2020.101398 32835933

[176] Sherif, A., Al-Sokary, E., Rizk, W. & Mahfouz, M. Immune status of *Oreochromis niloticus* subjected to long-term lead nitrate exposure and a *Arthrospira platensis* treatment trial. *Environmental Toxicology and Pharmacology* **76**, (2020). 10.1016/j.etap.2020.103352 32045721

[177] Shi, L. *et al*. Hematogenesis Adaptation to Long-Term Hypoxia Acclimation in Zebrafish (*Danio rerio*). *Fishes* **7**, (2022).

[178] Silkin, Y., Silkina, E., Silkin, M. & Chernyaeva, V. The Dimensional and Physiological-Biochemical Parameters of Erythrocytes and Gill and Heart Indices in Some Benthic Fish of the Black Sea Coast of the Southeastern Crimea. *Russian Journal of Marine Biology* **49**, 191–199 (2023).

[179] Silkin, Y., Silkina, E., Silkin, M. & Vasilets, V. The hematological parameters of the Black Scorpionfish *Scorpaena porcus* Linnaeus, 1758 under Experimental hypothermia *in vivo*. *Russian Journal of Marine Biology* **50**, 33–42 (2024).

[180] Simeon, E., Adaunwo, E. & Simeon, E. Effect of Paraquat on Organ Indices and Heamatology in *Clarias gariepinus* after Chronic Exposure. *British Journal of Pharmaceutical Research* **3**, 1106–1114 (2013).

[181] Singh, D., Nath, K., Trivedi, S. & Sharma, Y. Impact of copper on haematological profile of freshwater fish, *Channa punctatus*. *Journal of Environmental Biology* **29**, 253–257 (2008). 18831385

[182] Small, S. A. & Benfey, T. J. Cell size in triploid salmon. *Journal of Experimental Zoology* **241**, 339–342 (1987).

[183] Soldatov, A. Peculiarities of osmoregulation of circulating red blood cells in steno- and euryhaline marine fish species under hypoosmotic conditions. *Journal of Evolutionary Biochemistry and Physiology* **36**, 52–58 (2000).

[184] Soldatov, A. *et al*. The Functional Morphology of Erythrocytes of the Black Scorpion Fish *Scorpaena porcus* (Linnaeus, 1758) (Scorpaeniformes: Scorpaenidae) during Hypoxia. *Russian Journal of Marine Biology* **43**, 368–373 (2017).

[185] Suljevic, D. & Mitrasinovic-Brulic, M. The first record of brook trout (*Salvelinus fontinalis*, Salmonidae) blood cell characteristics and hematological profile: the influence of fish sex on leukocyte count. *Aquaculture International* **28**, 2505–2516 (2020).

[186] Talukdar, B. *et al*. Effects of Acid Mine Drainage of Coal Mines on Some Haematological Parameters of *Channa punctatus* (Bloch). *National Academy Science Letters-India* **40**, 91–94 (2017).

[187] Tang, Y. *et al*. Characterization of hematological parameters and blood cells of cultured *Gymnocypris eckloni* Herzenstein, 1891. *Journal of Applied Ichthyology* **31**, 931–936 (2015).

[188] Tavares-Dias, M., Moraes, F. & Imoto, M. Hematological parameters in two neotropical freshwater teleost, *Leporinus macrocephalus* (Anostomidae) and *Prochilodus lineatus* (Prochilodontidae). *Bioscience Journal* **24**, 96–101 (2008).

[189] Tort, L. & Torres, P. The effects of sublethal concentrations of cadmium on hematological parameters in the dogfish, *Scyliorhinus canicular*. *Journal of Fish Biology* **32**, 277–282 (1988).

[190] Trofimov, D. & Zabotkina, E. Effect of Trypanosome Infection on Hematological Parameters of the Black Sea Sprat (*Clupeonella cultriventris*) from the Ivankovo Reservoir. *Inland Water Biology* **17**, 370–373 (2024).

[191] Ullah, M. *et al*. Effect of Cypermethrin on Blood Hematology and Biochemical Parameters in Fresh Water Fish *Ctenopharyngodon idella* (Grass Carp). *Cellular and Molecular Biology* **68**, 15–20 (2022). 37114278 10.14715/cmb/2022.68.10.3 PMC10155271

[192] Ullah, S. *et al*. Bifenthrin induced toxicity in *Ctenopharyngodon idella* at an acute concentration: A multi-biomarkers based study. *Journal of King Saud University Science* **34**, (2022).

[193] Ural, M. Chlorpyrifos-induced changes in oxidant/antioxidant status and haematological parameters of *Cyprinus carpio carpio*: Ameliorative effect of lycopene. *Chemosphere* **90**, 2059–2064 (2013). 23312461 10.1016/j.chemosphere.2012.12.006

[194] Valdebenito, I., Busse, K., Jaramillo, N. & Hernández, A. Blood cytology of the common jollytail (*Galaxias maculatus*) (Jenyns, 1842) (Osmeriformes: Galaxiidae) at postlarval and adult stages. *Archivos de Medicina Veterinaria* **43**, 233–239 (2011).

[195] Volpato, G., Barreto, R., Marcondes, A., Moreira, P. & Ferreira, M. Fish ladders select fish traits on migration - still a growing problem for natural fish populations. *Marine and Freshwater Behaviour and Physiology* **42**, 307–313 (2009).

[196] Walencik, J. & Witeska, M. The effects of anticoagulants on hematological indices and blood cell morphology of common carp (*Cyprinus carpio* L.). *Comparative Biochemistry and Physiology C-Toxicology & Pharmacology* **146**, 331–335 (2007). 10.1016/j.cbpc.2007.04.004 17509941

[197] Wang, B., Liu, Y., Chen, X. & Fan, Z. Amitosis-like nuclear division in erythrocytes of triploid rainbow trout *Oncorhynchus mykiss*. *Journal of Fish Biology* **76**, 1205–1211 (2010). 20409171 10.1111/j.1095-8649.2010.02556.x

[198] Weinberg, S. R., Siegel, C. D. & Gordon, A. S. Studies on the peripheral blood cell parameters and morphology of the red paradise fish, *Macropodus opercularis*. Effect of food deprivation on erythropoiesis. *The Anatomical Record* **175**, 7–13 (1973). 4682842 10.1002/ar.1091750103

[199] Williams, E. & Eddy, F. Regulation of blood hemoglobin and electrolytes in rainbow trout *Salmo gairdneri* (Richardson) exposed to nitrite. *Aquatic Toxicology* **13**, 13–27 (1988).

[200] Wintrobe, M. M. Variations in the size and hemoglobin content of erythrocytes in the blood of various vertebrates. *Folia haematologica* **51**, 32–49 (1934).

[201] Witeska, M., Kondera, E., Lipionoga, J. & Jastrzebska, A. Changes in oxygen consumption rate and red blood parameters in common carp *Cyprinus carpio* L. after acute copper and cadmium exposures. *Fresenius Environmental Bulletin* **19**, 115–122 (2010).

[202] Wolters, W., Chrisman, C. & Libey, G. Erythrocyte nuclear measurements of diploid and triploid channel catfish, *Ictalurus punctatus* (Rafinesque). *Journal of Fish Biology* **20**, 253–258 (1982).

[203] Xiong, W. *et al*. Effects of temperature on metabolic scaling in silver carp. *Journal of Experimental Zoology Part A* **337**, 141–149 (2022). 10.1002/jez.2542 34492171

[204] Yao, T. *et al*. Effects of dietary supplementation with astragalus *(Astragalus membranaceus)* root or leaf meal on the hematology, serum biochemical parameters, histomorphology, oxidative status, and resistance of *Phoxinus lagowskii* against bacterial infection. *Aquaculture* **565**, (2023).

[205] Ye, X. *et al*. Metabolic scaling: individual versus intraspecific scaling of Nile tilapia (*Oreochromis niloticus*). *Journal of Comparative Physiology B-Biochemical Systemic and Environmental Physiology* **191**, 721–729 (2021). 33934186 10.1007/s00360-021-01376-8

[206] Zehra, S. & Khan, M. A. Effects of different levels of dietary cyanocobalamin on growth, liver cyanocobalamin concentration, antioxidant capacity, intestinal enzymes and non-specific immune response for optimum inclusion in the commercial feeds of fingerling *Channa punctatus* (Bloch). *Aquaculture* **511**, (2019).

[207] Zexia, G. *et al*. Morphological studies of peripheral blood cells of the Chinese sturgeon, *Acipenser sinensis*. *Fish Physiology and Biochemistry* **33**, 213–222 (2007).

[208] Zhang, H. *et al*. Haematological and blood biochemical characteristics of *Glyptosternum maculatum* (Siluriformes: Sisoridae) in Xizang (Tibet). *Fish Physiology and Biochemistry* **36**, 797–801 (2010). 19757131 10.1007/s10695-009-9354-2

[209] Zhang, H. J., Xie, C. X., Li, D. P., Liu, H. P. & Yang, X. F. Blood cells of a sisorid catfish *Glyptosternum maculatum* (Siluriformes: Sisoridae), in Tibetan Plateau. *Fish Physiology and Biochemistry* **37**, 169–176 (2011). 20737208 10.1007/s10695-010-9429-0

[210] Zhang, Y. *et al*. Intraspecific mass scaling of metabolic rates in grass carp (*Ctenopharyngodon idellus*). *Journal of Comparative Physiology B* **184**, 347–354 (2014). 10.1007/s00360-014-0802-7 24481482

[211] Zhu, D. *et al*. Flow cytometric determination of genome size for eight commercially important fish species in China. *In Vitro Cellular and Developmental Biology-Animal* **48**, 507–517 (2012). 22956044 10.1007/s11626-012-9543-7

[212] Zutshi, B., Prasad, S. & Nagaraja, R. Alteration in hematology of *Labeo rohita* under stress of pollution from Lakes of Bangalore, Karnataka, India. *Environmental Monitoring and Assessment* **168**, 11–19 (2010). 19603276 10.1007/s10661-009-1087-2

[213] Benfey, T. J. & Sutterlin, A. M. The haematology of triploid landlocked Atlantic salmon, *Salmo salar* L. *Journal of Fish Biology* **24**, 333–338 (1984).

[214] Martins, B. O., Franco-Belussi, L., Siqueira, M. S., Fernandes, C. E. S. & Provete, D. The evolution of red blood cell shape in fishes. 10.6084/m9.figshare.12044325.v3 (2020). 10.1111/jeb.13757 33484056

[215] Lenoir, J. *et al*. Species better track climate warming in the oceans than on land. *Nature Ecology & Evolution* **4**, 1044–1059 (2020). 32451428 10.1038/s41559-020-1198-2

[216] Leiva, F. P., Verberk, W. C. E. P., Calosi, P., Rezende, E. L. & Mark, F. C. MetaR, a global database on metabolic rates of ectotherms. Preprint at 10.32942/X2Z022 (2024).

[217] Ahyong, S. *et al*. World Register of Marine Species (WoRMS). WoRMS Editorial Board (2024).

[218] Leiva, F. P. *et al*. ShareTrait: Towards interoperable and reusable individual trait-based data in ectotherms. *Functional Ecology* **39**, 3124–3138 (2025).

[219] Rees, J. A. & Cranston, K. Automated assembly of a reference taxonomy for phylogenetic data synthesis. *Biodivers Data J* e12581 10.3897/BDJ.5.e12581 (2017). 10.3897/BDJ.5.e12581 PMC5515096 28765728

[220] Mozzherin, D. servis, Matt, Pereira, H. L. & wkollernhm. GlobalNamesArchitecture/biodiversity: v5.3.4. *Zenodo*10.5281/zenodo.5610073 (2021).

[221] R Development Core Team. R: A language and environment for statistical computing. R Foundation for Statistical Computing, Vienna, Austria (2023).

[222] Casajus, N. rutils: A collection of R functions commonly used in FRB-CESAB projects. R package version 0.0.0.9000 (2023).

[223] Wickham, H. & Bryan, J. readxl: Read Excel Files. R package version 1.4.3 (2023).

[224] Wickham, H., Francois, R., Henry, L., Müller, K. & Vaughan, D. dplyr: A Grammar of Data Manipulation. R package version 1.1.4 (2023).

[225] Wickham, H. The split-apply-combine strategy for data analysis. *Journal of statistical software* **40**, 1–29 (2011).

[226] Ooms, J. writexl: Export Data Frames to Excel ‘xlsx’ Format. R package version 1.5.0 (2024).

[227] Müller, K. & Wickham, H. tibble: Simple Data Frames. R package version 3.2.1 (2023).

[228] Wickham, H., Chang, W., Flight, R., Müller, K. & Hester, J. sessioninfo: R Session Information. R package version 1.2.2 (2021).

[229] Massicotte, P. & South, A. rnaturalearth: World Map Data from Natural Earth. R package version 1.0.1 (2023).

[230] Cambon, J., Hernangómez, D., Belanger, C. & Possenriede, D. tidygeocoder: An R package for geocoding. *JOSS* **6**, 3544 (2021).

[231] Zhu, H. kableExtra: Construct Complex Table with ‘kable’ and Pipe Syntax. R package version 1.4.0 (2024).

[232] Cui, B. DataExplorer: Automate Data Exploration and Treatment. R package version 0.8.3 (2024).

[233] McLean, M. W. RefManageR: Import and Manage BibTeX and BibLaTeX References in R. *JOSS* **2**, 338 (2017).

[234] McLean, M. W. Straightforward Bibliography Management in R with the RefManageR Package. Preprint at http://arxiv.org/abs/1403.2036 (2014).

[235] Chamberlain, S. *et al*. rgbif: Interface to the Global Biodiversity Information Facility API. R package version 3.7.8. (2024).

[236] Chamberlain, S. A. & Boettiger, C. *R Python, and Ruby Clients for GBIF Species Occurrence Data*. https://peerj.com/preprints/3304/ (2017).

[237] Boettiger, C., Lang, D. T. & Wainwright, P. C. rfishbase: exploring, manipulating and visualizing FishBase data from R. *Journal of Fish Biology* **81**, 2030–2039 (2012). 23130696 10.1111/j.1095-8649.2012.03464.x

[238] Chamberlain, S. & Szöcs, E. taxize: taxonomic search and retrieval in R. *F1000Research* **2**, (2013). 10.12688/f1000research.2-191.v1 PMC3901538 24555091

[239] Chamberlain, S. *et al*. taxize: Taxonomic information from around the web. R package version 0.9.98. (2020).

[240] Michonneau, F., Brown, J. W. & Winter, D. J. rotl: an R package to interact with the Open Tree of Life data. *Methods in Ecology and Evolution* **7**, 1476–1481 (2016).

[241] Paradis, E. & Schliep, K. ape 5.0: an environment for modern phylogenetics and evolutionary analyses in R. *Bioinformatics* **35**, 526–528 (2019). 30016406 10.1093/bioinformatics/bty633

[242] Revell, L. J. phytools 2.0: an updated R ecosystem for phylogenetic comparative methods (and other things). *PeerJ* **12**, e16505 (2024). 38192598 10.7717/peerj.16505 PMC10773453

[243] Yu, G. Using ggtree to visualize data on tree-like structures. *Current protocols in bioinformatics* **69**, e96 (2020). 32162851 10.1002/cpbi.96

[244] Yu, G., Smith, D. K., Zhu, H., Guan, Y. & Lam, T. T.-Y. ggtree: an R package for visualization and annotation of phylogenetic trees with their covariates and other associated data. *Methods in Ecology and Evolution* **8**, 28–36 (2017).

[245] Yu, G., Lam, T. T.-Y., Zhu, H. & Guan, Y. Two methods for mapping and visualizing associated data on phylogeny using ggtree. *Molecular biology and evolution* **35**, 3041–3043 (2018). 30351396 10.1093/molbev/msy194 PMC6278858

[246] Xu, S. *et al*. *Ggtree*: A serialized data object for visualization of a phylogenetic tree and annotation data. *iMeta* **1**, e56 (2022). 38867905 10.1002/imt2.56 PMC10989815

[247] Yu, G. *Data Integration, Manipulation and Visualization of Phylogenetic Trees*. (Chapman and Hall/CRC, 2022).

[248] Wickham, H. *Ggplot2: Elegant Graphics for Data Analysis*. (Springer-Verlag, New York, 2016).

[249] Kassambara, A. ggpubr: ‘ggplot2’ Based Publication Ready Plots. R package version 0.6.0. (2023).

[250] Schiettekatte, N., Brandl, S. & Casey, J. fishualize: Color Palettes Based on Fish Species. R package version 0.2.3. (2024).

[251] Wilke, C. O. cowplot: Streamlined plot theme and plot annotations for ‘ggplot2’. R package version 1.1.3. (2024).

[252] Arnold, J. ggthemes: Extra Themes, Scales and Geoms for ‘ggplot2’. R package version 5.0.0. (2023).

[253] Leiva, F. P. *et al*. ErythroCite: a database on red blood cell size of fishes. *Zenodo*10.5281/zenodo.18543381 (2026). 10.1038/s41597-026-06904-1 PMC12949179 41760678

[254] Molina‐Venegas, R. How to get the most out of phylogenetic imputation without abusing it. *Methods Ecol Evol* **15**, 456–463 (2024).

[255] Bruggeman, J., Heringa, J. & Brandt, B. W. PhyloPars: estimation of missing parameters values using phylogeny. *Nucleic Acids Research* **37**, W179–W184 (2009). 19443453 10.1093/nar/gkp370 PMC2703881

[256] Adrian, G. J., Czarnoleski, M. & Angilletta, M. J. Flies evolved small bodies and cells at high or fluctuating temperatures. *Ecology and Evolution* **6**, 7991–7996 (2016). 27878071 10.1002/ece3.2534 PMC5108251

[257] Czarnoleski, M., Labecka, A. M. & Kozłowski, J. Thermal plasticity of body size and cell size in snails from two subspecies of *Cornu aspersum*. *Journal of Molluscan Studies* **82**, 235–243 (2015).

[258] Kierat, J., Szentgyörgyi, H., Czarnoleski, M. & Woyciechowski, M. The thermal environment of the nest affects body and cell size in the solitary red mason bee (*Osmia bicornis* L.). *Journal of thermal biology* **68**, 39–44 (2017). 28689719 10.1016/j.jtherbio.2016.11.008

[259] van de Pol, I. L., Flik, G. & Verberk, W. Triploidy in zebrafish larvae: Effects on gene expression, cell size and cell number, growth, development and swimming performance. *PloS one* **15**, e0229468 (2020). 32119699 10.1371/journal.pone.0229468 PMC7051096

[260] Snyder, G. K. & Sheafor, B. A. Red blood cells: centerpiece in the evolution of the vertebrate circulatory system. *American zoologist* **39**, 189–198 (1999).

[261] Hessen, D. O., Daufresne, M. & Leinaas, H. P. Temperature-size relations from the cellular-genomic perspective. *Biological Reviews* **88**, 476–489 (2013). 23551915 10.1111/brv.12006

[262] Verberk, W. C. E. P. *et al*. Body mass and cell size shape the tolerance of fishes to low oxygen in a temperature-dependent manner. *Global Change Biology* **28**, 5695–5707 (2022). 35876025 10.1111/gcb.16319 PMC9542040

[263] Pottier, P., Burke, S., Drobniak, S. M., Lagisz, M. & Nakagawa, S. Sexual (in)equality? A meta-analysis of sex differences in thermal acclimation capacity across ectotherms. *Functional Ecology* **35**, 2663–2678 (2021).

[264] Pottier, P. *et al*. A comprehensive database of amphibian heat tolerance. *Scientific Data* **9**, 600 (2022). 36195601 10.1038/s41597-022-01704-9 PMC9532409

